# Spatiotemporal patterns of gliosis and neuroinflammation in presenilin 1/2 conditional double knockout mice

**DOI:** 10.3389/fnagi.2022.966153

**Published:** 2022-09-14

**Authors:** Wenjun Peng, Yuan Xie, Chongzheng Liao, Yunxia Bai, Huimin Wang, Chunxia Li

**Affiliations:** ^1^Key Laboratory of Brain Functional Genomics (STCSM and MOE), Affiliated Mental Health Center (ECNU), School of Psychology and Cognitive Science, East China Normal University, Shanghai, China; ^2^Shanghai Changning Mental Health Center, Shanghai, China; ^3^NYU-ECNU Institute of Brain and Cognitive Science at NYU Shanghai, Shanghai, China

**Keywords:** presenilins 1/2 conditional double knockout mice, neuroinflammation, gliosis, chemokine, Ccl3 and Ccl4

## Abstract

Increasing evidence indicates that neuroinflammation contributes to and exacerbates the pathogenesis of Alzheimer’s disease (AD). Neuroinflammation is thought to be primarily driven by glial cells (microglia and astrocytes) and escalates with neurodegenerative progression in AD. However, the spatiotemporal change patterns of glial reactivity and neuroinflammatory response during different stages of neurodegeneration, especially early in disease, remain unknown. Here we found that gliosis and the up-regulation of substantial neuroinflammatory genes were primarily initiated in the cortex of presenilin 1/2 conditional double knockout (cDKO) mice, rather than in the hippocampus. Specifically, astrocyte activation preceding microglial activation was found in the somatosensory cortex (SS) of cDKO mice at 6 weeks of age. Over time, both astrocyte and microglial activation were found in the whole cortex, and age-related increases in gliosis activation were more pronounced in the cortex compared to hippocampus. Moreover, the age-associated increase in glial activation was accompanied by a gradual increase in the expression of cell chemokines Ccl3 and Ccl4, complement related factors C1qb, C3 and C4, and lysosomal proteases cathepsin S and Z. These findings suggest that astrocyte and microglial activation with a concurrent increase in inflammatory mediators such as chemokines might be an early event and contribute to the pathogenesis of neurodegeneration due to presenilin deficiency.

## Introduction

Alzheimer’s disease (AD) is one of the most common neurodegenerative diseases in the elderly and has no effective neurocentric-based therapies. Individuals with AD are typically divided into early-onset and late-onset AD. Early onset AD, with symptoms appearing at a young age, is predominantly inherited with heritability estimates at 90% (Wingo et al., 2012). Mutations of the essential components of the γ secretase complex presenilin 1 (PS1), presenilin 2 (PS2) gene, and of the amyloid precursor protein (APP) gene, have been identified as important familial AD-causing mutations (Campion et al., 1999; Nussbaum and Ellis, 2003). Although early-onset familial AD only accounts for about 4–6% of total AD cases (Zhu et al., 2015), PS and APP gene mutations are widely believed to be critically involved in AD pathogenesis. Thus, studying the functions of PS will help decipher the biological mechanisms leading to AD pathology.

To study the function of PS in the cortex and the hippocampus and avoid possible compensation for the loss of either of the two proteins, PS1 and PS2 conditional double knockout (PS cDKO) mice lacking both presenilins in the postnatal forebrain were generated (Feng et al., 2004; Saura et al., 2004). PS cDKO mice exhibited AD-related pathology, including progressive memory impairment and synaptic dysfunction, tau pathology, as well as gliosis and robust inflammatory responses, followed by age-dependent forebrain neurodegeneration, which can occur without amyloid-β (Aβ) deposition (Beglopoulos et al., 2004; Feng et al., 2004; Saura et al., 2004; Jiang et al., 2009; Wines-Samuelson et al., 2010; Zhang et al., 2010). Specific postsynaptic defects were first found in PS cDKO mice at 6 weeks of age (Zhang et al., 2010) and memory impairment was detected at 2 months (Saura et al., 2004). At 3 months, significant increase in strong inflammatory responses was observed in the cortex of cDKO mice (Dong et al., 2007). Loss of cortical neurons and volume was not found at 2 months but it was found with progressive severity at 4 months of age (Wines-Samuelson et al., 2010). At 5 months, increased astrocyte activation and hyperphosphorylated of tau were seen in the cortex of cDKO mice (Cao et al., 2018). Severe impairment of learning and memory was accompanied by a dramatic increase in astrogliosis, microgliosis and inflammatory responses and cortical shrinkage in PS cDKO mice at 6 months of age (Saura et al., 2004; Jiang et al., 2009). A recent work has suggested that clinical PS mutations may cause familial AD through a loss-of-function mechanism (Xia et al., 2015). Unlike various Aβ-depositing models such as APP/PS1 transgenic mouse models (Drummond and Wisniewski, 2017), PS cDKO mice lacked amyloid aggregation but recapitulated key features of AD, and presented with significant early-onset neuroinflammation, which preceded the appearance of other neurodegenerative symptoms. Thus, PS cDKO mice provide us with a good model for dissection of the early events and neuroinflammatory pathways leading to progressive memory deficits and neurodegeneration.

Neuroinflammation is thought to be involved in a vicious cycle of glial priming, release of proinflammatory chemokines, neuronal damage in the earliest stages of AD (Calsolaro and Edison, 2016). Numerous recent studies have indicated that glial cells mediated neuroinflammation play prominent roles in driving the pathogenesis and progression of AD (Li et al., 2011; Heppner et al., 2015; Van Eldik et al., 2016; Chun et al., 2020). Genome-wide association studies have identified risk variants of some immune genes and found associations between mutations in these genes and AD (Guerreiro et al., 2013; Thambisetty et al., 2013; Efthymiou and Goate, 2017). Recent findings have not only identified microglial and astrocyte activation before the onset of Aβ deposition in AD mouse model and gliosis in the brain of individuals with AD or prodromal forms of AD (Heneka et al., 2005; Carter et al., 2012; Johnson et al., 2020) but have also found increased levels of inflammatory cytokines, chemokines and related mediators in patients with mild cognitive impairment (MCI) and AD (Brosseron et al., 2014; Heppner et al., 2015), suggesting that glial cells and their mediated inflammatory responses may be an early feature in the pathogenesis and progression of AD.

Previous studies demonstrated that various treatments, including environment enrichment, administration of histone deacetylase inhibitors and anti-inflammatory drugs ameliorated the memory deficits of PS cDKO mice through suppressing gliosis and inflammatory activation (Dong et al., 2007; Cao et al., 2018; Zhao et al., 2019). Although glial activation and neuroinflammatory responses have been proposed to be an important process in neurodegeneration, the precise temporal and spatial spectrum of the onset of astrocyte and microglial activation and other immune related changes were less known in PS cDKO mice. In the current study, using 6-week-old, and 2–4-month-old PS cDKO mice, we aimed to reveal the relationship of astrocyte and microglial activation to neurodegeneration initiation and progression.

## Materials and methods

### Mice

PS cDKO mice were generated and genotyped as described previously (Feng et al., 2004). Briefly, PS cDKO mice were obtained by crossing the forebrain specific PS1 heterozygous knockout mice with conventional PS2 knockout (PS2^–/–^) mice on B6/CBA genetic background. Mice with the Cre transgene, fPS1/fPS1, and PS2^–/–^ served as PS cDKO mice, their littermates (without Cre, fPS1/^+^ and PS2^+/+^) served as wild-type (WT) controls. Both male and female PS cDKO mice were used in the experiments. Mice had *ad libitum* access to water and chow and were housed at 22 ± 2 °C in 12 h/12 h light/dark schedule and 50–60% humidity. The experiment protocols were approved by the Animal Ethics Committee of East China Normal University.

### Western blotting

Cortical and hippocampal tissue were obtained from 2-, 3-, 4-month-old mice, experiments were carried out as described previously with minor modifications (Cao et al., 2018). Briefly, mice were decapitated and the head was immersed in liquid nitrogen for 4 s, then the cortex and hippocampus were quickly dissected. Total protein was prepared from both tissues as described previously (Cao et al., 2018) and the protein concentration was measured with a BCA kit (Beyotime, China). 60 μg of proteins was boiled with SDS loading buffer and separated on 5–15% SDS-PAGE gels and transferred onto a PVDF membrane. The membrane was blocked with 5% skim milk in TBST for 1 h at room temperature, incubated with primary antibodies in 5% skim milk/TBST at 4 °C overnight, and then rinsed 4 times with TBST. The membrane was then incubated with secondary antibodies for 1 h at room temperature, and images were captured by Odyssey LI-COR Imager. The primary antibodies used include rabbit-anti-GFAP (1:10,000, abcam); mouse-anti-GAPDH (1:5,000, bioworld); rabbit-anti-IBA1 (1:1,000, abcam). The secondary antibodies used include anti-Mouse IRDye 800CW (1:15,000, Li-Cor Biosciences, United Kingdom) and anti-Rabbit IRDye 800CW (1:15,000, Li-Cor Biosciences, United Kingdom). The immunoreactive bands were quantified by the ImageJ software. Band intensities were normalized to loading control GAPDH (anti-GAPDH antibody, 1:5,000, Bioworld, United States).

### Immunofluorescence

Mice were deeply anesthetized and perfused transcardially with ice-cold 4% paraformaldehyde (PFA) in PBS and brains were fixed in 4% PFA at 4 °C overnight. Frozen brain blocks were sliced into 20 μm sections using a cryostat (Leica Microsystems, Germany). The sections were subsequently washed for 15 min with 1.2% Triton X-100 in PBS, blocked in blocking buffer (5% normal goat serum, 2% BSA and 0.2% Triton X-100) for 1 h, and incubated with primary antibodies at 4 °C overnight. After washing with 0.1% Tween 20 in PBS, the sections were incubated with secondary antibodies for 2 h at room temperature. The sections were mounted with antifade mounting medium (Beyotime, China) and imaged using Zeiss Axio Imager A1 microscope (Carl Zeiss Microscopy GmbH, Germany). The primary antibodies used: rabbit-anti-GFAP (1:1,000, abcam, ab7260); rabbit-anti-IBA1 (1:1,000, abcam, ab178847). The secondary antibodies used: Alexa Fluor^®^ 488 Goat Anti-Rabbit IgG (H + L) Antibody (1:600, Invitrogen). The number and positive area of GFAP^+^ or IBA1^+^ cells were measured using the ImageJ analysis software.

### Ribonucleic acid sequencing

For RNA Sequencing (RNA-Seq), cortex samples from two mice were pooled to generate one sample (*n* = 3). Total RNA was extracted by Trizol Reagent (Invitrogen Life Technologies, United States), and RNA quality was determined using NanoDrop spectrophotometer (Thermo Scientific, United States). After RNA purification, the sequencing library was constructed and then sequenced on a Navaseq 6000 sequncing platform (Illumina, United States) by Personalbio Co., Ltd. (Shanghai, China).

The raw data obtained by sequencing were filtered by removing adaptor-related and low-quality reads, and then the clean reads were mapped to the mouse reference genome. The expression level of each gene was calculated and expressed by the FPKM value (Fragments Per Kilobase of exon per Million fragments mapped). DESeq was used for analysis of differentially expressed genes (DEGs), and genes with a | log_2_ (fold change) | > 1 and *p*-value < 0.05 were considered differentially expressed. The Gene Ontology (GO) and Kyoto Encyclopedia of Genes and Genomes (KEGG) pathway enrichment analysis of DEGs were conducted as described previously, respectively (Ashburner et al., 2000; Kanehisa et al., 2004). GO and Pathways were considered significantly enriched with *p* < 0.05. The Protein-Protein Interaction (PPI) network was analyzed using the online STRING database,^1^ which provides physical (direct) interactions and functional (indirect) associations between proteins. Based on the DEGs, the PPI pairs with score > 0.95 were identified from the STRING database. After the PPI analysis by STRING, the interaction network was plotted and visualized by Cytoscape software.

### Real time polymerase chain reaction

Real time polymerase chain reaction (RT-PCR) was performed as described previously (Cao et al., 2018). Briefly, cortex and hippocampus of cDKO mice and WT mice were dissected and immediately frozen in liquid nitrogen and stored in a freezer at -80 °C prior to use. RNA was extracted using Trizol (Invitrogen, United States) and cDNA was generated using moloney murine leukemia virus (MMLV) reverse transcriptase (Invitrogen, United States). Diluted cDNA was used as a template for the TB Green (Takara, RR420) RT-PCR analysis using CFX97 real time system (Bio-Rad, United States). The primer sequences for RT-PCR are listed in Supplementary Table 2. Glyceraldehyde-3-phosphate dehydrogenase (GAPDH) was used as the reference gene. The gene expression levels were calculated and described as 2^–Δ^ ^ΔCt^ values.

### Statistical analyses

All data were presented as mean ± SEM. To compare data between two groups, unpaired two-tailed *t*-test was used. To examine significance among more than two groups, one-way ANOVA followed by Tukey’s multiple comparisons test were used. All statistical analyses were performed using GraphPad Prism. Statistical significance was set at *p* < 0.05 for all tests.

## Results

### Progressive increase of gliosis in presenilin 1/2 conditional double knockout mice

**FIGURE 1:**
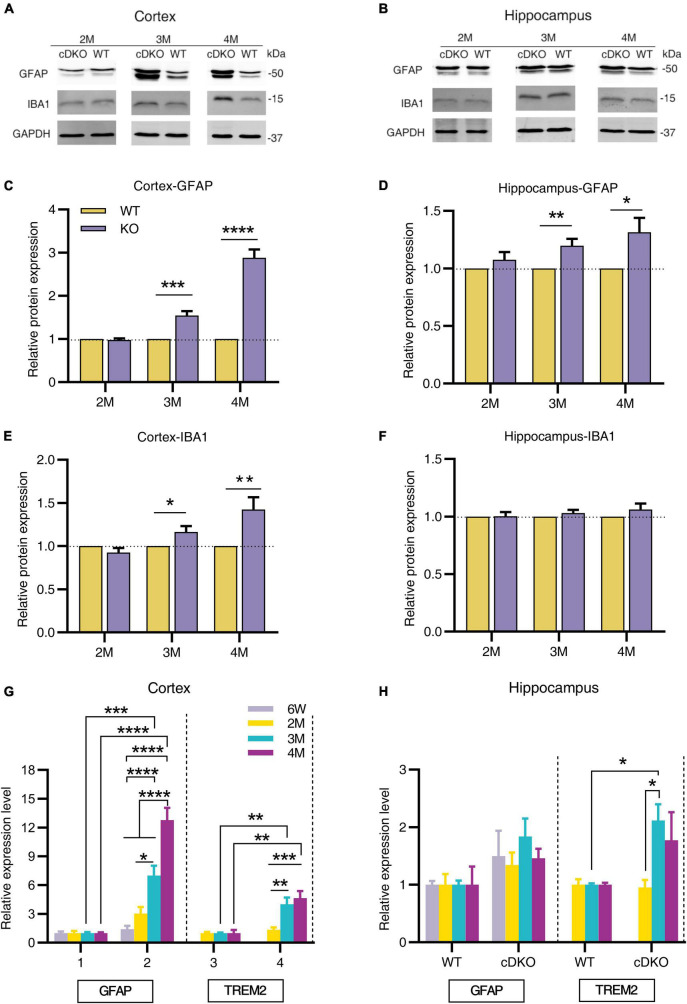
Progressive increase in protein and mRNA expression of glial genes in cDKO mice. **(A,B)** Representative Western blots of astrogliosis marker GFAP and microgliosis marker IBA1 in the cortex **(A)** and hippocampus **(B)** from the 2-, 3-, 4-month-old (2, 3 and 4 M) WT and cDKO mice. **(C–F)** Quantification of relative levels of GFAP and IBA1 as normalized to GAPDH protein expression (Cortex, 2 M: *n* = 6; 3 M: *n* = 7; 4 M: *n* = 8; Hippocampus, 2 M: *n* = 3; 3 M: *n* = 6; 4 M: *n* = 6). Data are presented as the mean ± SEM. *P < 0.05, **p < 0.01, ***p < 0.001, ****p < 0.0001, unpaired two tailed *t*-test. **(G,H)** Relative mRNA expression of GFAP and TREM2 normalized to GAPDH in the cortex **(G)** and hippocampus **(H)** of WT and cDKO mice at the ages of 6 weeks, 2, 3, and 4 months analyzed by RT-PCR. Data are presented as the mean ± SEM (*n* = 3–5 for each group). One-way ANOVA analysis followed by Turkey’s test, *p < 0.05, **p < 0.01, ***p < 0.001, ****p < 0.0001.

To further investigate the dynamic patterns of glial cells in different brain regions of cDKO mice with age, we first used Western blotting to determine the protein levels of astrogliosis marker glial fibrillary acidic protein (GFAP) and microgliosis marker ionized calcium-binding adapter molecule 1 (IBA1) in the cortex and hippocampus of cDKO mice at 2, 3, and 4 months. At 2 months of age we found that both GFAP and IBA1 did not change their protein expression level in the cortex and hippocampus (Figures 1A–F), there was a significant increase in GFAP expression in both cortex and hippocampus of 3- and 4-month-old cDKO mice (Figures 1A–D), whereas we only detected a higher IBA1 expression in the cortex without a different expression in the hippocampus of 3- and 4-month-old cDKO mice (Figures 1A,B,E,F). We further performed real time-PCR (RT-PCR) to probe the expression changes of GFAP and triggering receptor expressed on myeloid cells 2 (TREM2). As shown in Figure 1G, significantly increased GFAP expression was found at 3 and 4 months, revealing age-dependent and progressive upregulation in the cortex of cDKO mice. Similarly, we also found a significant increase in the expression of TREM2, a microglial surface immunoreceptor, in the cortex of cDKO mice (Figure 1G). However, in the hippocampus, the expression of GFAP was comparable to controls at all ages examined (Figure 1H), but a significant increase in TREM2 expression was observed in cDKO mice at 3 months (Figure 1H). Thus, cDKO mice exhibited age-related, progressive increase of gliosis with significantly more GFAP expression in the cortex.

### Increase of astrogliosis, but not microgliosis in the somatosensory cortex of conditional double knockout mice at 6 weeks and 2 months of age

**FIGURE 2:**
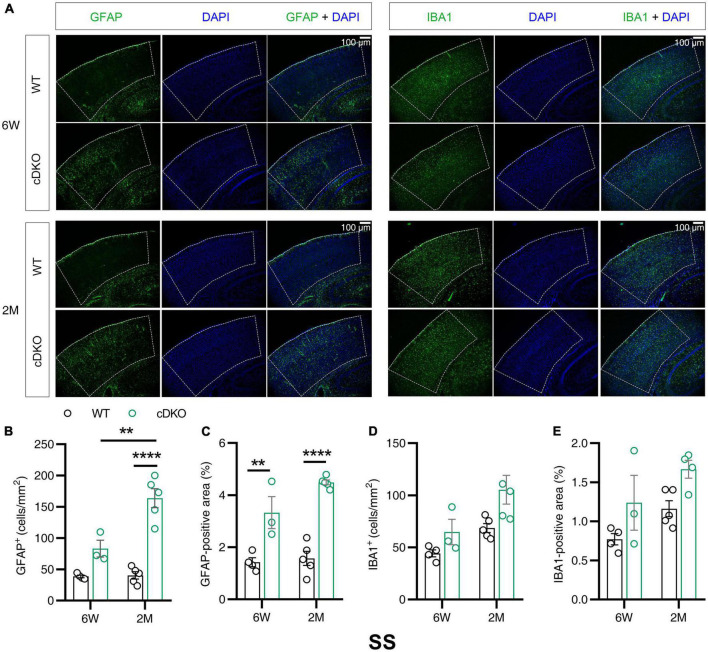
Astrocyte reactivity, but not the microglial reactivity was increased in the somatosensory cortex (SS) of cDKO mice at 6 weeks of age. **(A)** Representative images of immunohistochemistry for GFAP and IBA1 of WT (control) and cDKO brains at 6 weeks (6 W) and 2 months (2 M) of age. **(B–E)** Quantification of GFAP-positive cells **(B)**, GFAP-positive areas **(C)**, IBA1-positive cells **(D)** and IBA1-positive areas **(E)** in the SS of WT and cDKO mice. Data are presented as the mean ± SEM (*n* = 3–5 for each group). One-way ANOVA analysis followed by Turkey’s test, **p < 0.01, ****p < 0.0001. Scale bar 100 μm.

Western blotting results revealed that the protein expression levels of GFAP and IBA1 increased with age in the cortex and hippocampus at 3 and 4 months of age. Notably, PS cDKO mice exhibited impaired post-synaptic responses as early as 6 weeks old prior to the onset of neurodegeneration (Zhang et al., 2010). The subregions of cortex reported to be affected in the very early stage of AD include somatosensory cortex (SS) and retrosplenial cortex (RS) (Pengas et al., 2010; Stephen et al., 2010). Given that Western blotting examined the changes in the whole cortex or hippocampus, we performed immunofluorescence experiments to gain insight into whether in the subregions of the cortex and hippocampus astrocyte and microglial activation occurred in the early stage before the onset of neurodegeneration. We measured GFAP and IBA1 immunoreactivity in 6-week- and 2-month-old cDKO mice. In the SS of cDKO mice, GFAP reactivity increased at both 6 weeks and 2 months of age (Figures 2A–C). However, there was no significant change in number or positive area (%) of microglia between cDKO mice and age-matched controls (Figures 2A,D,E). Thus, the occurrence of astrogliosis appeared to start earlier than that of microgliosis in the SS of cDKO mice.

### Increase of astrogliosis and microgliosis in the retrosplenial cortex of presenilin 1/2 conditional double knockout mice at 6 weeks and 2 months

**FIGURE 3:**
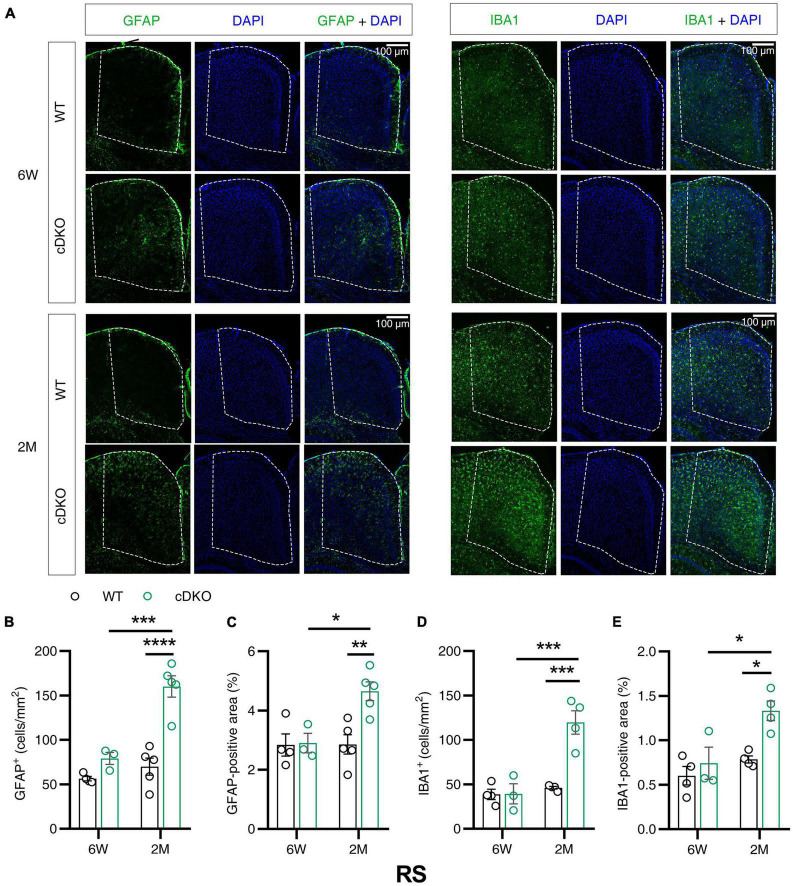
Astrocyte reactivity and microglial reactivity were increased in the retrosplenial cortex (RS) of PS cDKO mice at 2 months of age. **(A)** Representative images of immunohistochemistry for GFAP and IBA1 of WT (control) and cDKO brains at 6 weeks (6 W) and 2 months (2 M) of age. **(B–E)** Quantification of GFAP-positive cells **(B)**, GFAP-positive areas **(C)**, IBA1-positive cells **(D)** and IBA1-positive areas **(E)** in the RS of WT and cDKO mice. Data are presented as the mean ± SEM (*n* = 3–5 for each group). One-way ANOVA analysis followed by Turkey’s test, *p < 0.05, **p < 0.01, ***p < 0.001, ****p < 0.0001. Scale bar 100 μm.

Given that the volume loss and atrophy of retroslenial cortex (RS) was observed at the earliest clinical stage of AD (Pengas et al., 2010), we further examined GFAP and IBA1 immunoreactivity in the RS of cDKO mice at 6 weeks and 2 months of age. At 6 weeks, no detectable difference was found in GFAP and IBA1 reactivity of the RS in the cDKO mice relative to control (Figures 3A–E). Comparison of stained brain sections and quantitative analysis revealed a striking increase in the number and positive area of astrocyte and microglia in the RS of cDKO mice at 2 months of age, whereas we could easily see age-dependent and progressive increases of both astrogliosis and microgliosis in the RS of cDKO mice (Figures 3A–E).

### Normal astrogliosis and microgliosis in the hippocampus of presenilin 1/2 conditional double knockout mice at 6 weeks and 2 months

We next investigated whether astrocyte and microglia reactivity changed in the hippocampus of cDKO mice. At 6 weeks and 2 months, for all subregions of the hippocampus, including CA1, CA3 and dentate gyrus (DG), there were no statistically significant differences in GFAP reactivity between cDKO and WT mice (Supplementary Figures 1A–C). Similarly, IBA1 reactivity was also unaltered in cDKO mice relative to WT mice at both 6 weeks and 2 months of age (Supplementary Figures 2A–C).

### Characterizing the transcriptomics of the cortex in 2-month-old presenilin 1/2 conditional double knockout mice

**FIGURE 4:**
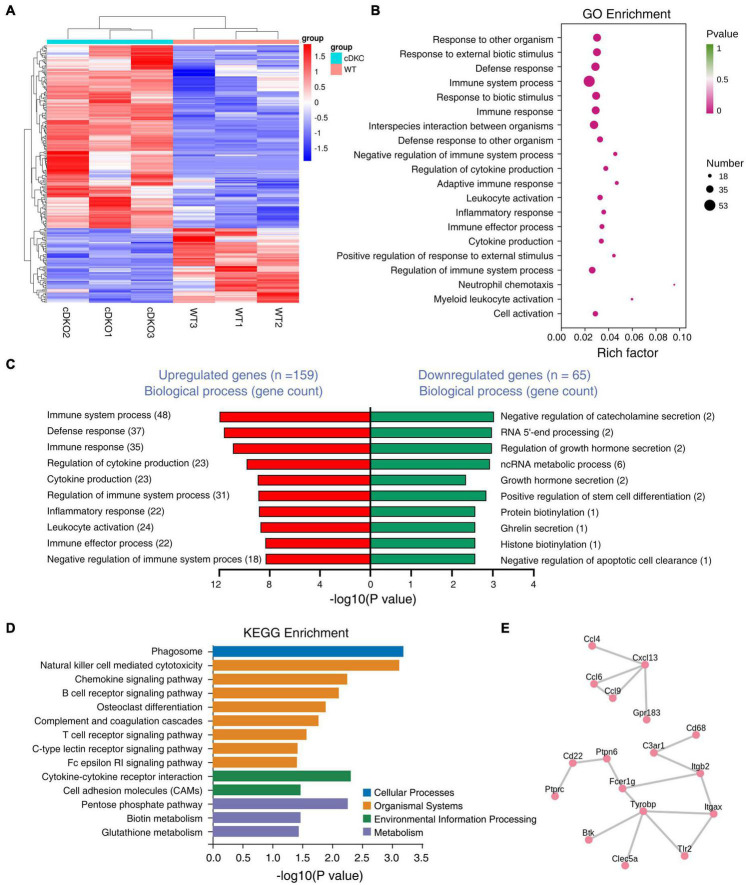
Differentially expressed genes (DEGs) in the cortex of 2-month-old PS cDKO mice. **(A)** RNA sequencing data in a heatmap and hierarchical clustering. The 159 up- and 65 down-regulated DEGs were used to generate the heatmap. Upregulated genes are indicated in red, downregulated genes are indicated in blue, *n* = 3 per group. **(B,C)** GO enrichment analysis of DEGs from 2M animals. Bubble plots showing the top 20 GO biological processes **(B)** and the top GO biological processes for DEGs upregulated and downregulated are ranked by the number of gene counts **(C)**. **(D)** Kyoto Encyclopedia of Genes and Genomes (KEGG) pathways enrichment analysis for DEGs upregulated in the cortex of cDKO mice compared with control mice. Distribution of the KEGG pathway categories were assigned into four categories: cellular processes, organismal systems, environmental information processing and metabolism. **(E)** Protein-protein interactions (PPIs) network analysis of DEGs in the cortex of cDKO mice.

To characterize what changes in gene expression are potentially associated with the gliosis in the cortex of cDKO mice, we performed transcriptome sequencing analysis using RNA from the cortex of 2-month-old cDKO mice to identify DEGs. According to the fold change (> 2) and *p*-value (<0.05) criteria, a total of 224 DEGs were differentially regulated, of which 159 DEGs were up-regulated and 65 DEGs were down-regulated (Figure 4A and Supplementary Table 1). Notably, we found that glial and inflammatory related genes include astrocyte marker GFAP, inflammatory chemokines Ccl3, Ccl4, Ccl6, and Ccl9 and complement C1qb were all upregulated in 2-month-old of cDKO mice (Supplementary Table 1). Intriguingly, RNA-seq results of 2-month-old cDKO mice have both confirmed and further expanded previous microarray and RNA-seq results of cDKO mice at 3, 5, 6, 10 months of age (Beglopoulos et al., 2004; Dong et al., 2007; Cao et al., 2018), suggesting an early involvement of gliosis and inflammation in early neurodegeneration due to presenilins deficiency.

We next used gene ontology (GO) enrichment analysis to determine cellular processes and pathways affected in the cDKO mice and identified top 20 enriched GO terms (Figure 4B). Enriched biological process GO terms were related to immune system process and regulation, defense response, inflammatory related process, chemokine production and its regulation (Figure 4C). We found that downregulated genes were mostly related to generalized cellular functions. In contrast, upregulated genes were more associated with inflammation related process.

To obtain a deeper insight into the functions of upregulated DEGs, KEGG pathway analysis was performed on the 159 DEGs. The top-one in KEGG pathway analysis was phagosome, which is involved in homeostasis, innate and adaptive immune responses (Russell et al., 2009; Figure 4D). Interestingly, enriched KEGG pathway also included chemokine signaling pathway, cytokine-cytokine receptor interaction, complement and coagulation cascades (Figure 4D). Collectively, KEGG enrichment analysis of upregulated DEGs revealed an association of presenilins deficiency with phagocytosis and inflammatory cytokines and chemokines. KEGG enrichment analysis was also consistent with GO term analysis that revealed immune/inflammatory related processes, cytokine production and its regulation as the top-two enriched biological process GO term (Figures 4B,C).

To further identify the pivotal genes involved in the onset and progression of early neurodegeneration, DEGs were used to construct the PPI. Importantly, we identified two key PPI networks, an immune response and microglia activation related gene network (Tyrobp, Fcer1g, Cd68, C3ar1, Tlr2, Itgax, Itgb2, Ptprc, Cd22, Ptpn6, Btk, and Clec5a) and a cytokine/chemokine related gene network including Ccl4, Ccl6, Ccl9, Cxcl13, and Gpr183 (Figure 4E). Some enriched genes including Tyrobp and C3ar1 were selected for RT-PCR analysis to validate the RNA-seq data. RT-PCR results confirmed these genes were upregulated, as observed from the RNA-seq data (Supplementary Figure 3). Consistent with biological process GO term enrichment results, proteins encoded by these genes in these two networks are highly related to inflammation (Figure 4C).

### Spatiotemporal expression patterns of glial and inflammation-related genes in presenilin 1/2 conditional double knockout mice

Given the gliosis observed in the early neurodegeneration of cDKO mice, we further determined whether increased gliosis was accompanied by the corresponding changes in the expression of inflammatory factors with age in different brain regions of cDKO mice. We performed RT-PCR to probe the expression changes of several representative DEGs chose based on RNA-seq results and previous microarray and RNA-seq data in the cortex and the hippocampus of 6-week-, 2-, 3-, and 4-month-old cDKO mice. These included five inflammatory chemokines (Ccl2-Ccl6), four complement genes (C1qa, C1qb, C3, and C4) and four members of the cathepsin (Cath) family (Cath B, D, S, and Z).

**FIGURE 5:**
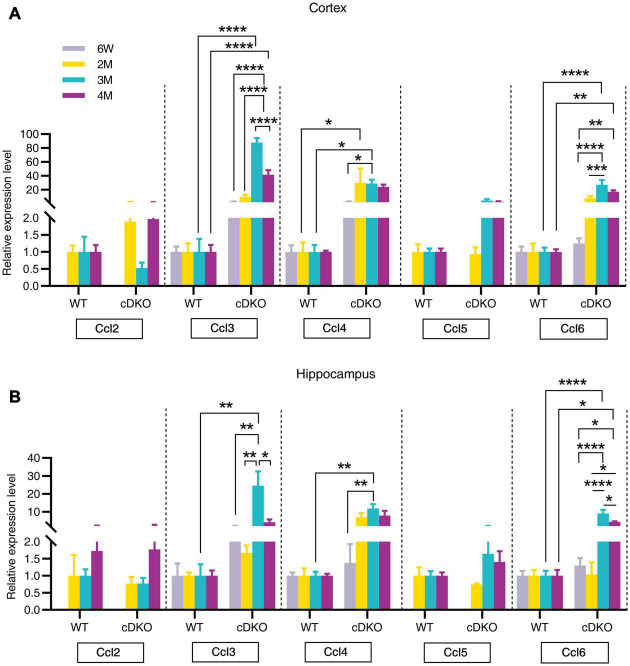
Age-related increase in mRNA expression of chemokine genes in the cortex and hippocampus of cDKO mice. **(A,B)** Relative expression of chemokine gene Ccl2, Ccl3, Ccl4, Ccl5, and Ccl6 normalized to GAPDH in the cortex **(A)** and hippocampus **(B)** of WT and cDKO mice at the ages of 6 weeks, 2, 3, and 4 months analyzed by RT-PCR. Data are presented as the mean ± SEM (*n* = 3–5 for each group). One-way ANOVA analysis followed by Turkey’s test, *p < 0.05, **p < 0.01, ***p < 0.001, ****p < 0.0001.

Chemokines, which are proinflammatory or chemotactic cytokines, are important mediators in inflammation and have been potentially implicated in contributing to AD pathogenesis (Lalli et al., 2015; Marciniak et al., 2015). Here, we detected an age-dependent increase of Ccl3, Ccl4, and Ccl6 expression with no alterations in Ccl2 and Ccl5 expression within both the cortex and hippocampus of cDKO mice (Figures 5A,B). Interestingly, we found that Ccl4 mRNA level was highly increased in the cortex of cDKO mice compared with littermate controls at 2 months (Figure 5A), appearing much earlier than the upregulation of other Ccl genes, suggesting that Ccl4 upregulation may accompany or precede astrocyte and microglia activation and is likely an early event anticipating the onset and progression of neurodegeneration.

**FIGURE 6:**
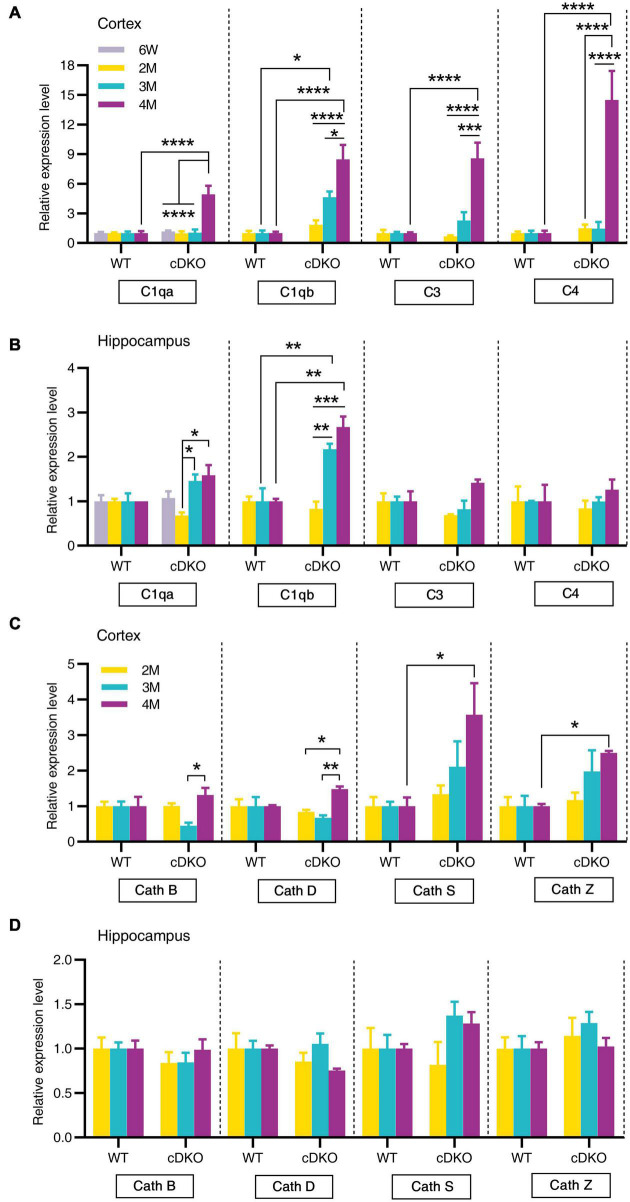
Age-related increase in mRNA levels of complement genes and cathepsin genes in the cortex and hippocampus of cDKO mice. **(A,B)** Relative expression of complement C1qa, C1qb, C3, and C4 normalized to GAPDH in the cortex **(A)** and hippocampus **(B)** of WT and cDKO mice at the ages of 6 weeks, 2, 3, and 4 months analyzed by RT-PCR. **(C,D)** Relative expression of cathepsin (Cath) B, D, S and Z normalized to GAPDH in the cortex **(C)** and hippocampus **(D)** of WT and cDKO mice at the ages of 6 weeks, 2, 3, and 4 months analyzed by RT-PCR. Data are presented as the mean ± SEM (*n* = 4–5 for each group). One-way ANOVA analysis followed by Turkey’s test, *p < 0.05, **p < 0.01, ***p < 0.001, ****p < 0.0001.

Since the classical complement cascade including C1q, C3, and C4 can be produced by glia and is involved in AD attributable to neuroinflammation (Dalakas et al., 2020), we next examined the expression of these complement genes changes with age. Indeed, striking increases in complement genes C1qa, C3, and C4 were seen in the cortex of cDKO mice at 4 months but not at 2 and 3 months (Figure 6A). Interestingly, no increase was found in C1qa, C3, and C4 expression in the hippocampus of cDKO mice, relative to controls (Figure 6B), whereas significant increases of C1qb expression were found in both the cortex and hippocampus of cDKO mice at 3 and 4 months of age, compared to the control (Figures 6A,B). Notably, despite the large increases in C1qb expression in both the cortex and hippocampus of cDKO mice, the increase of C1qb expression in cDKO mice was much more prominent in the cortex (Figure 6A).

We next assessed whether members of the cathepsin protease family (Cath B, D, S and Z), which are endo-lysosomal proteases and have been implicated in AD pathogenesis (Lemere et al., 1995; Di Domenico et al., 2016), showed age-dependent and progressive changes in expression in cDKO mice. Significant increases of Cath S and Cath Z expression were also seen in the cortex of cDKO mice at 4 months but not at 2 and 3 months compared with controls (Figure 6C). The increase of these cathepsin genes in the cDKO hippocampus was not significant relative to controls (Figure 6D), suggesting that cathepsin dysfunction may be a later event in the onset and progression of neurodegeneration in cDKO mice.

## Discussion

The prominent neuroinflammation marked by glial activation and increased levels of proinflammatory chemokines and cytokines has been postulated to contribute to the pathogenesis of AD (Heneka et al., 2015; Heppner et al., 2015; Calsolaro and Edison, 2016; Sorrentino et al., 2021). Changes in microglia and astrocyte are evident in patients with AD or MCI, as well as in animal models (Fukuyama et al., 2001; Carter et al., 2012; Heneka et al., 2015). Increasing evidence further supports an early involvement of neuroinflammation in AD pathogenesis based on the analysis of patients with MCI, which usually precedes AD (Okello et al., 2009; Carter et al., 2012; Winston et al., 2019). Thus, activation of microglia and astrocyte might occur in the early stage of AD, even before Aβ plaques formation (Heneka et al., 2005). However, the spatiotemporal pattern of glial activation and dysregulation of inflammatory mediators before the emergence of memory impairment or initiation of neurodegeneration remains largely unknown.

The current study demonstrates that in presenilins deficient mice the activation of microglia and astrocyte in the cortex happens earlier than in the hippocampus. Interestingly, this increase in astrocyte and microglial activation we have identified in 2-month-old cDKO mice has expanded upon previous findings that significant increase of degenerating neurons was present in the cortex, but not in the hippocampus, beginning at 2 months of age followed by age-related, progressive loss of cortical neurons in cDKO mice (Wines-Samuelson et al., 2010). Further, we also observed that the increase of astrocyte activation occurred relatively early compared with microglial activation in cDKO mice at 6 weeks of age, at least in SS. However, both astrocyte and microglia activation occurred simultaneously in RS in cDKO mice at 2 months, suggesting glial activation was heterologously present in different cortical subareas. Intriguingly, microglia in familial AD patient carrying mutation in PS1 gene who died at the young age were homogeneously distributed in the frontal cortex with a greater IBA1 protein levels and a higher cellular density compared to the AD patient carrying APPA673V mutations, which exhibited a specific distribution of microglia around amyloid plaques (Sorrentino et al., 2021). Whether microglia activation observed in the current study plays key roles in pathogenesis of neurodegeneration in presenilins deficient mice and whether microglial activation might be related to or triggered by astrocyte activation requires further investigation. Despite these findings, age-related and progressive increases of both astrocyte and microglial reactivity were robust in cDKO mice, suggesting that the activation of these cells may accurately reflect the onset and progression of neurodegeneration.

To identify the cellular players and molecular signals underlying the increased glial activation, we performed transcriptomic analysis of the cortex from 2-month-old cDKO mice. We identified 224 DEGs, 38 immune and inflammatory related genes of which all were exclusively upregulated (Supplementary Table 1) and accounted for 24% of all upregulated genes in the cortex of cDKO mice. GO and KEGG enrichment analyses of the DEGs comparing cDKO mice with control mice revealed enrichment of upregulated genes involved in immune and inflammatory processes and downregulated genes mainly related to metabolism and homeostasis (Figures 4B–D), which is consistent with previous finding that impaired brain energetics is associated with astrocyte and microglia activation in asymptomatic AD and that these early changes in energy metabolism and inflammation may play important roles in the progression of asymptomatic AD to AD (Johnson et al., 2020). These closely related processes and pathways imply that in the cDKO mice, astrogliosis and microgliosis may trigger and interact with neuroinflammation leading to initiation and progression of neurodegeneration (Heneka et al., 2014, 2015).

We also found that elevated expression of TREM2 occurred in both the cortex and the hippocampus of cDKO mice at 3 and 4 months of age. TREM2 variants have been identified as AD risk genes (Guerreiro et al., 2013; Jonsson et al., 2013). Even though its protective and harmful roles during the pathogenesis of AD remain incompletely defined (Jay et al., 2015; Wang et al., 2015; Zhong et al., 2019), increasing evidence suggest that TREM2 is closely related to microgliosis, astrogliosis, neuroinflammation, neuronal loss and cognition impairment in AD (Jay et al., 2017). Interestingly, PPI network analysis identified Tyrobp, a key regulator in immune and inflammatory systems, to be one of the major hub genes. As a signaling adaptor of TREM2, Tyrobp is mainly expressed in microglia, mutated in early-onset AD (Pottier et al., 2016), increased its expression in the AD brains (Haure-Mirande et al., 2017) and may be associated with early-onset AD. However, a recent study has indicated that Tyrobp–Apoe pathway could be an initiating step in transforming of the disease associated microglia phenotype and upregulation of Tyrobp in recruited microglia around amyloid plaques and traumatic lesion is TREM-independent (Audrain et al., 2021). Therefore, pathological interaction between TREM2-Tyrobp signaling and gliosis, as well as neuroinflammation in prodromal AD should continue to be explored in the future.

Intriguingly, our RNA-seq results are similar to those with Aβ-depositing APPswe/PS1L166P transgenic mice, which exhibited very early amyloid deposition beginning at approximately 6 weeks in the cortex and 3–4 months in the hippocampus, amyloid associated astrocytosis and microgliosis starting from approximately 4 months of age, and memory deficits by the age of 7–8 months (Drummond and Wisniewski, 2017; Sierksma et al., 2020). Using APPswe/PS1L166P transgenic mice, Sierksma et al. identified many upregulated genes involved in astrocyte (GFAP), microglia and neuroinflammation, such as Tyrobp, Itgax and Cst7 (Sierksma et al., 2020). Single microglia sequencing further confirmed increased expression of Ccl3, Ccl4, and Clec7a in hippocampal activated response microglia cells of APPswe/PS1L166P mice (Sierksma et al., 2020). In the current study, upregulation of GFAP, Tyrobp, Itgax, Cst7, Ccl3, Ccl4, and Clec7a were also observed in no Aβ-depositing PS cDKO mice (Figure 4E and Supplementary Table 1). Overall, these findings suggest that the gliosis and neuroinflammatory responses, which are already present even before memory alterations appear, in both APP/PS1 transgenic mice and PS cDKO mice are fundamentally similar despite exhibiting very different Aβ phenotypes. It remains to be elucidated the underlying mechanisms and whether these glia and neuroinflammation related genes we identified in the current study are detrimental to neurodegeneration progression.

Chemokines, which are mainly produced by neurons, microglia and astrocytes in the brain, have been suggested to regulate microglial migration to local inflammatory sites and enhance inflammation in AD (Heneka et al., 2015). The current study provided an important comparison of spatiotemporal expression patterns of chemokine genes during the neurodegeneration of cDKO mice. Here, our RT-PCR confirmed an upregulation of Ccl3, Ccl4, and Ccl6 expression in both the cortex and hippocampus of cDKO mice at 3 and 4 months of age. Recent studies have indicated that astrocyte is an important source of Ccl3 and suggested a role for Ccl3 in the orchestration of microglial recruitment and activation (Cudaback et al., 2015), and cognitive function (Marciniak et al., 2015). Specially, Ccl4 was also upregulated in the cortex at 2 months. Ccl4 has been detected predominantly in reactive astrocytes of AD brains (Xia et al., 1998). Our findings suggest that Ccl4 may play key roles in astrocytes and be used as an early indicator of preclinical AD. In addition, we also observed upregulation of C1qb in both the cortex and the hippocampus of cDKO mice at 3 and 4 months of age, whereas C3 and C4 were upregulated in the cortex at 4 months. Recent studies have implicated the possible pathogenic roles of astrocytes in AD: inflammatory complement proteins C1q, C3d and C4b in astrocyte-derived exosomes are elevated in AD patients (Goetzl et al., 2018), and plasma astrocyte-derived exosome levels of these complement proteins may be used for prediction of MCI to AD conversion (Winston et al., 2019). Together, these data suggest that complement C1qb as well as chemokines Ccl3, Ccl4, and Ccl6 may mediate the capacity of astrocytes and microglia to damage neurons in the early stage of neurodegeneration (Winston et al., 2019). However, direct link between a chemokine change and a behavior is still unknown. In addition, it should also be noted that RNA-seq results are derived from whole cells.

## Conclusion

In summary, we have performed a comparative and comprehensive analysis of cDKO mice at 6 weeks, 2, 3, and 4 months of age to investigate brain area- and age-related changes of neuroinflammation in the early stages of the neurodegenerative process. We found that presenilins deficiency results in earlier onset of increased astrocyte activation in the SS of 6-week-old cDKO mice, which is earlier than memory deficits, suggesting that neuroinflammation may be involved in early neurodegenerative processes and contribute to later memory impairment. Over time, astrocyte and microglia activation was present in the whole cortex, accompanied by significant upregulation of chemokines and complement components in an age-dependent and brain-area specific manner, reminiscent of the age-related and progressive neurodegeneration in AD. The current study provides experimental support for the idea (Heneka et al., 2015; Calsolaro and Edison, 2016; Kwon and Koh, 2020) that increased neuroinflammation is an underlying cause for the onset and progression of neurodegeneration in presenilins deficient mice. These findings suggest that modifying astrocyte and microglial activation and related inflammatory signaling in the earlier stages of neurodegeneration could be an attractive therapeutic strategy for preventing the development of neurodegeneration.

## Data availability statement

The datasets presented in this study can be found in online repositories. The names of the repository/repositories and accession number(s) can be found below: www.ncbi.nlm.nih.gov/bioproject/PRJNA870676.

## Ethics statement

The animal study was reviewed and approved by the Animal Ethics Committee of East China Normal University.

## Author contributions

WP performed experiments, analyzed data, and wrote the manuscript. CXL designed the experiments, helped with analyzing data, and wrote the manuscript. YX, CZL, and YB contributed to molecular and animal experiments. CXL and HMW supervised the project and revised the manuscript. All authors contributed to the article and approved the submitted version.

## Footnotes

1
http://cn.string-db.org/cgi/input.pl

## Abbreviations

AD: Alzheimer’s disease
cDKO: conditional double knockout
PS1: presenilin 1
PS 2: presenilin 2
APP: amyloid precursor protein
PS cDKO: presenilin 1/2 conditional double knockout
WT: wild-type
DEGs: differentially expressed genes
GO: Gene Ontology
KEGG: Kyoto Encyclopedia of Genes and Genomes
PPI: Protein-Protein Interaction
MMLV: moloney murine leukemia virus
GFAP: glial fibrillary acidic protein
IBA1: ionized calcium-binding adapter molecule 1
RT-PCR: real time-PCR
SS: somatosensory cortex
RS: retrosplenial cortex
DG: dentate gyrus
Cath: cathepsin.
