# Supplementary material for: Spatiotemporal patterns of gliosis and neuroinflammation in presenilin 1/2 conditional double knockout mice
Source: Front Aging Neurosci. 2022 Sep 14;14:966153. doi: 10.3389/fnagi.2022.966153 (PMC9521545; doi:10.3389/fnagi.2022.966153)
Supplement: Supplementary file 1 [file Data_Sheet_1.pdf]

Supplementary Material

1 Supplementary Figures

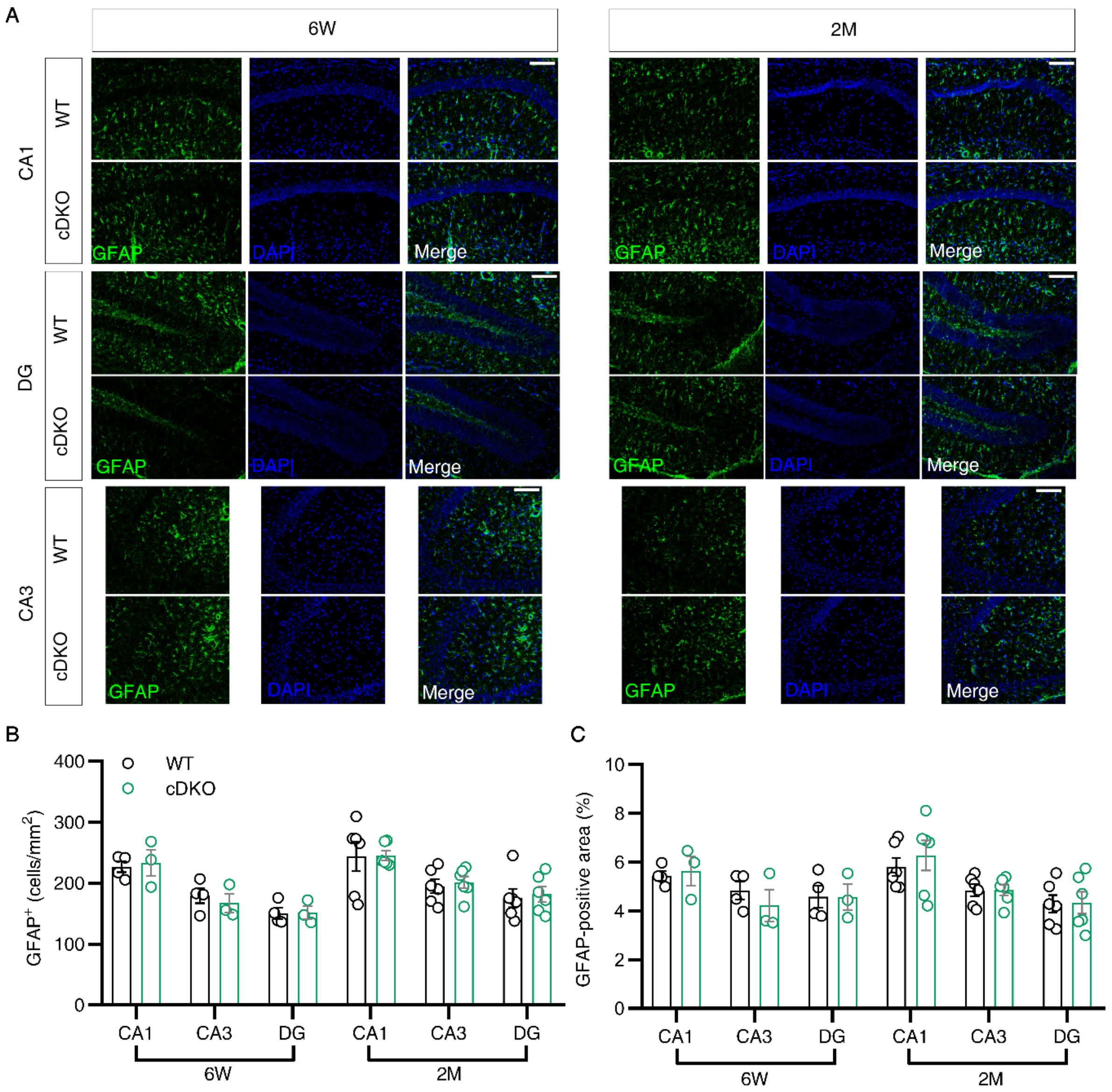

Supplementary Figure 1 Astrocyte reactivity was normal in the hippocampus of PS cDKO mice at 6 weeks and 2 months of age. **(A)** Representative images of immunohistochemistry for GFAP of control (WT) and cDKO brains at 6 weeks (6W) and 2 months (2M) of age. **(B-C)** Quantification of GFAP-positive cells **(B)** and GFAP-positive areas **(C)** in the hippocampal subregions CA1, CA3 and Dentate Gyrus (DG) of WT and cDKO mice. Data are presented as the mean ± SEM (n = 3–6 for each group). One-way ANOVA analysis followed by Turkey's test. Scale bar 50 µm.

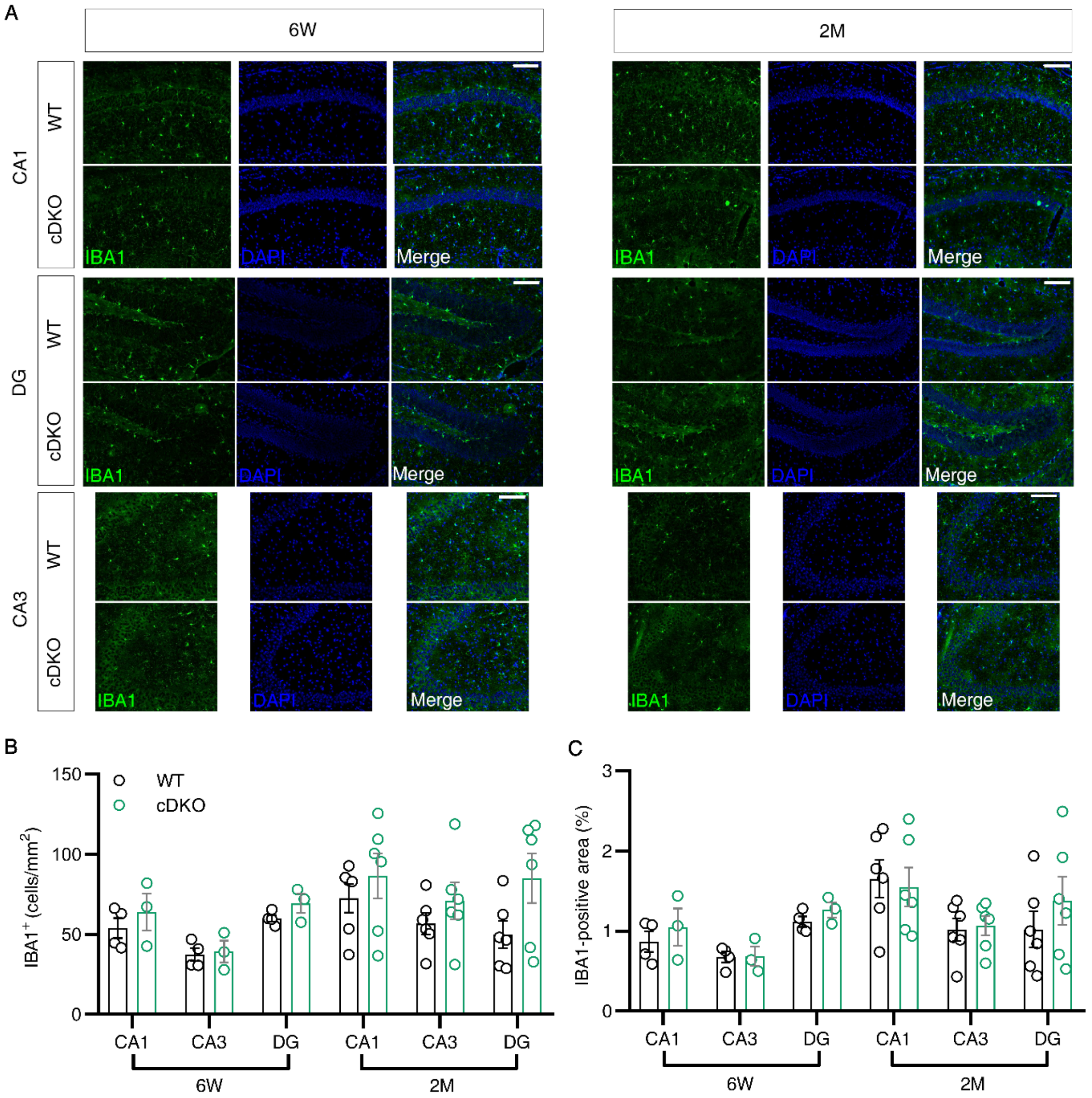

Supplementary Figure 2 Microglial reactivity was normal in the hippocampus of PS cDKO mice at 6 weeks and 2 months of age. **(A)** Representative images of immunohistochemistry for IBA1 of control (WT) and cDKO brains at 6 weeks (6W) and 2 months (2M) of age. **(B-C)** Quantification of IBA1-positive cells **(B)** and IBA1-positive areas **(C)** in the hippocampal subregions CA1, CA3 and Dentate Gyrus (DG) of WT and cDKO mice. Data are presented as the mean ± SEM (n = 3–6 for each group). One-way ANOVA analysis followed by Turkey's test. Scale bar 50 μm.

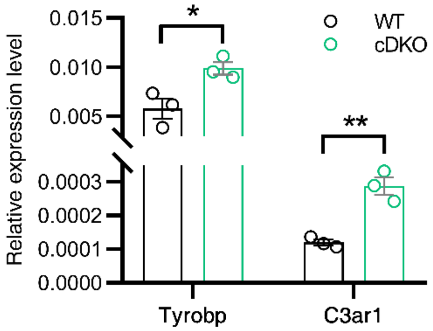

Supplementary Figure 3 mRNA expression levels of some enriched genes in the cortex of 2-month-old PS cDKO mice and WT mice. Relative expression of Tyrobp and C3ar1 normalized to GAPDH analyzed by RT-PCR. Data are presented as the mean ± SEM (n = 3 for each group). Two-tailed *t* test, *p < 0.05, **p < 0.01.

2 Supplementary Tables

Supplementary Table 1 The differentially expressed genes in the cortex of 2-month-old PS cDKO mice

Gene	baseMean_WT	baseMean_cDKO	Foldchange (cDKO/WT)	Regulation
Ctse	0	13.54718	Inf	Up Regulation
Dcdc2b	0	54.6535	Inf	Up Regulation
Ccl3	0	8.496657	Inf	Up Regulation
Cd22	0	7.728621	Inf	Up Regulation
Ccl4	0	10.7314	Inf	Up Regulation
Eqtn	0	8.736484	Inf	Up Regulation
Gm17330	0	4.479222	Inf	Up Regulation
LOC115490152	0	4.324802	Inf	Up Regulation
Mmp12	0	4.114892	Inf	Up Regulation
Hdac1	0.325058	132.2071	406.7191	Up Regulation
Iqcc	0.324736	77.91419	239.9307	Up Regulation
Eif3i	1.332486	226.0965	169.6802	Up Regulation
Mettl17	0.324736	47.60554	146.5976	Up Regulation
Cst7	0.682692	95.85757	140.4111	Up Regulation
Txlna	1.690442	222.0826	131.3754	Up Regulation
Clec7a	0.97453	84.24099	86.44268	Up Regulation
Ndrg2	18.75158	1032.665	55.07084	Up Regulation
Itgax	6.403171	213.642	33.36503	Up Regulation
Cxcl13	0.357635	11.81535	33.03747	Up Regulation
Adgrb2	51.57593	1659.075	32.16762	Up Regulation
Clcnka	0.324736	9.348908	28.78923	Up Regulation
Hist1h4n	0.324736	8.175767	25.17664	Up Regulation
Kpna6	11.63346	282.1741	24.2554	Up Regulation
Cryba2	0.357635	8.301389	23.21192	Up Regulation
LOC115487094	0.324736	7.01902	21.61453	Up Regulation
Marcksl1	11.47161	231.0777	20.14345	Up Regulation
Col16a1	6.109405	79.5976	13.0287	Up Regulation
Ccl6	11.24292	137.8232	12.25866	Up Regulation
Entpd4b	5.68758	67.59533	11.88473	Up Regulation
Khdrbs1	47.93314	460.5328	9.607815	Up Regulation
Siglecf	3.705621	34.41731	9.287865	Up Regulation
LOC115489454	1.689478	15.31929	9.067468	Up Regulation
Tmem39b	5.914263	49.48023	8.366254	Up Regulation
LOC115487110	2.339594	17.36822	7.423607	Up Regulation

Kif18b	1.007429	7.395401	7.340868	Up Regulation
Bfsp2	4.744341	32.73032	6.898812	Up Regulation
Tmem234	18.68764	122.7968	6.571018	Up Regulation
Pdcd1	1.755597	10.68768	6.087776	Up Regulation
Gpx6	1.040327	6.208737	5.968062	Up Regulation
Olfr464	1.365385	8.037461	5.88659	Up Regulation
Il1rl1	1.332165	7.617361	5.718032	Up Regulation
Slamf9	3.347344	19.03076	5.685331	Up Regulation
Cd180	10.20588	56.31282	5.517683	Up Regulation
Fgr	3.671758	18.97295	5.167267	Up Regulation
Gpr65	2.307016	11.85907	5.140435	Up Regulation
Slfn9	2.698192	13.70076	5.077753	Up Regulation
Aqp6	1.981316	9.626638	4.858709	Up Regulation
Arl11	2.340236	11.00857	4.704042	Up Regulation
Lrmp	6.566307	30.70752	4.676528	Up Regulation
Tlr2	9.782127	44.24236	4.522775	Up Regulation
C3ar1	17.22491	74.67096	4.335056	Up Regulation
Slc39a2	7.117476	29.82731	4.190715	Up Regulation
Casc1	5.233499	21.87708	4.180202	Up Regulation
Ncf4	4.321874	17.64743	4.083281	Up Regulation
Lag3	47.35133	190.3919	4.020836	Up Regulation
Per3	57.98679	220.954	3.81042	Up Regulation
Lyz2	79.58236	301.3951	3.78721	Up Regulation
Phc2	116.1164	438.333	3.774943	Up Regulation
Gfap	1583.353	5872.782	3.709079	Up Regulation
Lilrb4a	5.229321	19.33581	3.697576	Up Regulation
Rhod	3.640145	13.10066	3.598939	Up Regulation
Rep15	4.744984	17.05168	3.593621	Up Regulation
Eno1	1039.549	3705.401	3.564432	Up Regulation
Myo1f	36.3029	129.2307	3.559789	Up Regulation
Gpr15	7.540265	26.77465	3.55089	Up Regulation
Slc45a1	127.9378	436.0336	3.40817	Up Regulation
Ccl9	13.90886	46.99004	3.378425	Up Regulation
St14	5.00328	16.82362	3.362518	Up Regulation
Fgfr1op2	533.1485	1782.735	3.343788	Up Regulation
Tm7sf3	139.7488	465.7273	3.332604	Up Regulation
Rec8	10.85864	36.05483	3.320382	Up Regulation
Bcat1	347.0783	1109.993	3.198106	Up Regulation
Park7	194.7123	615.997	3.163626	Up Regulation
Cd52	11.53605	35.83175	3.106068	Up Regulation
Ints13	103.0148	319.6774	3.103219	Up Regulation

Tyrobp	90.76392	281.4293	3.100674	Up Regulation
Ccdc91	142.8651	434.871	3.043927	Up Regulation
Spx	11.76312	35.71929	3.036549	Up Regulation
LOC108167440	88.2035	265.7021	3.012376	Up Regulation
Ptp4a2	197.515	585.2751	2.963193	Up Regulation
Ly9	5.329302	15.72384	2.950449	Up Regulation
Pgd	34.7357	101.4557	2.920791	Up Regulation
Etfrf1	64.59127	187.4507	2.902106	Up Regulation
Lpo	4.128732	11.9442	2.892946	Up Regulation
Cd84	18.09657	52.32179	2.891255	Up Regulation
H6pd	21.79872	62.95406	2.88797	Up Regulation
Col6a4	11.57127	32.9581	2.848271	Up Regulation
Kras	437.5777	1236.629	2.826079	Up Regulation
Klhl42	170.2266	480.2182	2.821052	Up Regulation
Rpl21	173.1785	480.1377	2.772503	Up Regulation
Arntl2	50.84016	140.8609	2.770662	Up Regulation
Tent5c	8.806954	24.165	2.743855	Up Regulation
Slc15a3	15.30425	41.69255	2.724246	Up Regulation
Cecr2	5.426391	14.73384	2.715219	Up Regulation
Iqgap3	6.563414	17.6339	2.686697	Up Regulation
Ccdc28b	31.9791	85.85567	2.684743	Up Regulation
Apobec1	14.49061	38.53563	2.659352	Up Regulation
Pbk	5.428319	14.39859	2.652494	Up Regulation
Stk38l	133.7597	354.0348	2.646797	Up Regulation
Ptprc	46.60587	122.3563	2.625341	Up Regulation
Cd48	9.586665	25.10007	2.618227	Up Regulation
Scpep1	64.79062	168.9849	2.608169	Up Regulation
Cpsf4l	14.94887	38.96757	2.606725	Up Regulation
Ly86	77.79276	201.567	2.591076	Up Regulation
Slc25a18	194.7051	500.195	2.568987	Up Regulation
Cd68	88.70998	227.7262	2.567087	Up Regulation
Clec5a	13.7783	35.36729	2.566884	Up Regulation
Hbb-b2	56.96596	145.7957	2.559349	Up Regulation
Mks1	19.92839	50.49566	2.533856	Up Regulation
Cfap54	99.3449	251.4909	2.531493	Up Regulation
Wwox	312.29	788.0156	2.523346	Up Regulation
Btk	9.457712	23.80095	2.516565	Up Regulation
Inmt	6.660182	16.43491	2.467637	Up Regulation
Gm42048	20.07988	49.42033	2.461186	Up Regulation
Cd74	39.90629	98.04131	2.456788	Up Regulation

Efhc1	10.85471	26.647	2.454879	Up Regulation
Cyba	28.14246	68.83461	2.445935	Up Regulation
Mpeg1	238.2436	580.9878	2.438629	Up Regulation
Acadl	30.87201	75.24261	2.437244	Up Regulation
Llgl2	10.66021	25.78206	2.418532	Up Regulation
Lpar5	9.715366	22.95073	2.362313	Up Regulation
Ppm1m	126.7138	297.4465	2.347389	Up Regulation
Hbb-b1	311.5833	725.5811	2.328691	Up Regulation
Dmrt2	8.581235	19.92238	2.321622	Up Regulation
Morc2b	31.06997	71.90002	2.314132	Up Regulation
Mrps35	79.86462	184.023	2.304187	Up Regulation
Gkn3	18.55715	41.75981	2.250336	Up Regulation
Akr1e1	121.9412	268.0781	2.19842	Up Regulation
Lancl1	136.5143	299.5173	2.194036	Up Regulation
Coil	126.2648	276.7462	2.191792	Up Regulation
Gpr183	9.618599	21.02539	2.18591	Up Regulation
Akap1	223.2054	486.1401	2.177995	Up Regulation
Vps52	15.37005	33.35949	2.170422	Up Regulation
Thrsp	167.9577	362.4115	2.157754	Up Regulation
Tagap	47.63474	102.729	2.156599	Up Regulation
Nek11	10.78795	23.24458	2.15468	Up Regulation
Samsn1	19.20726	41.12924	2.141338	Up Regulation
Capg	16.28135	34.72671	2.132913	Up Regulation
C1qb	388.4777	825.2178	2.124235	Up Regulation
Zc3h12a	11.3742	24.07924	2.117006	Up Regulation
Itgb2	59.89294	126.209	2.107243	Up Regulation
Phex	12.64121	26.632	2.106761	Up Regulation
Rpe	24.76428	52.12485	2.10484	Up Regulation
9930012K11Rik	13.71282	28.62895	2.08775	Up Regulation
Lat2	26.03194	54.33282	2.08716	Up Regulation
Gpr137b	144.7752	301.1116	2.079856	Up Regulation
Cybrd1	21.08763	43.79207	2.076671	Up Regulation
Rpl29	598.1461	1230.926	2.057902	Up Regulation
Tnfrsf18	28.59596	58.73932	2.054112	Up Regulation
Tuba1c	353.2538	724.518	2.050984	Up Regulation
Cd164l2	20.0502	41.10191	2.04995	Up Regulation
Ptpn6	42.01977	85.79318	2.041733	Up Regulation
Msi2	682.034	1385.458	2.031362	Up Regulation
Fcer1g	62.26158	126.2798	2.028214	Up Regulation
Dgke	645.3728	1307.115	2.025364	Up Regulation
Tspoap1	1108.517	2243.897	2.024234	Up Regulation

Csf3r	93.78852	189.5579	2.02112	Up Regulation
Rdh12	12.63928	25.43504	2.012381	Up Regulation
Agmat	12.35105	24.73833	2.002934	Up Regulation
Zfp772	145.9513	72.78766	0.498712	Down Regulation
Casp9	674.4039	335.9187	0.498097	Down Regulation
Rims1	88.69833	42.74595	0.481925	Down Regulation
Slc22a2	20.31042	9.751141	0.480105	Down Regulation
Zdhhc8	85.176	39.55883	0.464436	Down Regulation
H2-T24	277.6202	126.5241	0.455745	Down Regulation
Hlcs	155.8541	68.59409	0.440117	Down Regulation
B3gnt6	16.08532	6.668493	0.41457	Down Regulation
Pigp	125.7513	51.55327	0.409962	Down Regulation
Ttc3	5904.178	2330.836	0.394777	Down Regulation
Cep170b	145.5961	55.63965	0.382151	Down Regulation
Il7r	22.42565	8.357792	0.372689	Down Regulation
Lsmem2	22.8158	8.397728	0.368066	Down Regulation
Gabbr1	241.1872	87.21777	0.361619	Down Regulation
Cubn	17.15461	5.998827	0.349692	Down Regulation
Sp7	24.46755	8.552707	0.349553	Down Regulation
Gm10265	18.68649	6.391322	0.342029	Down Regulation
Rpp21	52.35082	17.88263	0.341592	Down Regulation
Khdc3	18.62487	6.335272	0.340151	Down Regulation
Krt12	43.89893	14.23008	0.324156	Down Regulation
Olfr287	67.17227	20.96724	0.312141	Down Regulation
Gm14322	98.62178	30.72805	0.311575	Down Regulation
Piwil2	26.64651	8.245339	0.309434	Down Regulation
Gm3317	10.49836	3.221514	0.306859	Down Regulation
Sox5	115.1983	33.61109	0.291767	Down Regulation
Upk1b	12.28589	3.013356	0.24527	Down Regulation
Psen2	167.5833	40.83679	0.243681	Down Regulation
Zfp970	172.3995	41.90632	0.243077	Down Regulation
Ppp1r11	484.0629	113.7287	0.234946	Down Regulation
Gtf2h4	49.75414	11.62183	0.233585	Down Regulation
Gm5796	10.3987	2.39918	0.230719	Down Regulation
Nat8f6	19.00955	4.338605	0.228233	Down Regulation
Wfikkn1	8.708902	1.981112	0.227481	Down Regulation
Gm14325	278.2212	62.49073	0.224608	Down Regulation
Rpl34	311.556	62.51868	0.200666	Down Regulation
Ceacam16	10.04171	1.981112	0.197288	Down Regulation
H3f4	8.38288	1.590929	0.189783	Down Regulation

LOC100862433	17.83602	3.223266	0.180717	Down Regulation
Gm42346	7.150375	1.255956	0.175649	Down Regulation
Sh2d4a	7.733407	1.242154	0.160622	Down Regulation
Hist1h2bq	6.56245	1.032244	0.157295	Down Regulation
Jakmip3	34.54545	5.303308	0.153517	Down Regulation
Ghsr	6.434784	0.962671	0.149604	Down Regulation
Vars2	104.5881	15.52626	0.148451	Down Regulation
Cchcr1	19.98897	2.902376	0.145199	Down Regulation
Wdr46	7.309975	1.046046	0.143098	Down Regulation
Ccdc158	13.94112	1.96731	0.141116	Down Regulation
Vmn2r29	84.37071	11.06287	0.131122	Down Regulation
LOC102638085	5.654681	0.725156	0.12824	Down Regulation
Cd209g	5.979096	0.683468	0.11431	Down Regulation
Cd300lf	30.54631	3.181578	0.104156	Down Regulation
Lvrn	11.66764	1.004359	0.086081	Down Regulation
LOC100861615	17.35393	1.297644	0.074775	Down Regulation
Kazn	223.3866	16.49271	0.07383	Down Regulation
2310061I04Rik	116.804	7.171688	0.061399	Down Regulation
Gm12185	6.303511	0.362578	0.05752	Down Regulation
Farsb	130.1954	5.566956	0.042758	Down Regulation
Krt20	81.95332	2.5536	0.031159	Down Regulation
Catspere1	26.65701	0.307088	0.01152	Down Regulation
Utp14b	38.42624	0	0	Down Regulation
Cdsn	9.557051	0	0	Down Regulation
Spink4	5.004245	0	0	Down Regulation
Prap1	4.645967	0	0	Down Regulation
Gm38702	4.419284	0	0	Down Regulation
Hsd3b2	4.063256	0	0	Down Regulation

Supplementary Table 2 Primers used in RT-PCR analysis

Gene	Foward primer	Reverse primer
C1qa	5’-CGGGTCTCAAAGGAGAGAGA-3’	5’-TATTGCCTGGATTGCCTTTC-3’
C1qb	5’-CAGGGATAAAGGGGGAGAAA-3’	5’-TCTGTGTAGCCCCGTAGTCC-3’
TREM2	5’-GACCTCTCCACCAGTTTCTCC-3’	5’-TACATGACACCCTCAAGGACTG-3’
Ccl2	5’-TTAAAAACCTGGATCGGAACCAA-3’	5’-GCATTAGCTTCAGATTTACGGGT-3’
Ccl3	5’-CCTCTGTCACCTGCTCAACATCA-3’	5’-CATATGGCGCTGAGAAGACTTGG-3’
Ccl4	5’-TGCTCGTGGCTGCCTTCT-3’	5’-CTGCCGGGAGGTGTAAGAGA-3’
Ccl5	5’-GCTTGGGGATGCCACTCAGTAAT-3’	5’-CTGATTTCTTGGGTTTGCTGTGC-3’
Ccl6	5’-GCTGGCCTCATACAAGAAATGG-3’	5’-GCTTAGGCACCTCTGAACTCTC-3’
Cathepsin Z	5’-CCCTGGTGCTGCTGATGTT-3’	5’-TGACGCTGGCATAGTTGACAC-3’
Cathepsin D	5’-GGCGACCTCTGGCTTTAAGC-3’	5’-CTCCGTGGTCTTAGGCGATG-3’
Cathepsin S	5’-CTGTGGCAATGGAGCAACTG-3’	5’-AGTGACGCAGCCCTTCTCTC-3’
Cathepsin B	5’-TTGCGTTCGGTGAGGACATA-3’	5’-CTTCAGCAGACACCTCCACG-3’
C3	5’-AGCAGCACAGAAGAGACCAT-3’	5’-TGCCACACAGATCCCTTTCT-3’
C4	5’-ACTTCAGCAGCTTAGTCAGGG-3’	5’-GTCCTTTGTTTCAGGGGACAG-3’
GFAP	5’-CGGAGACGCATCACCTCTG-3’	5’-AGGGAGTGGAGGAGTCATTCG-3’
Tyrobp	5’-TGGTGTTGACTCTGCTGATTGC-3’	5’-CCTTCCGCTGTCCCTTGAC-3’
C3ar1	5’-TCGATGCTGACACCAATTCAA-3’	5’-TCCCAATAGACAAGTGAGACCAA-3’
Tlr2	5’-GCAAACGCT GTTCTGCTCAG-3’	5’-AGGCGTCTCCCTCTATTGTATT-3’
CD68	5’-ACTGGTGTAGCCTAGCTGGT-3’	5’-CCTTGGGCTATAAGCGGTCC-3’
GAPDH	5’-AGGAGCGAGACCCCACTAACAT-3’	5’-GTGATGGCATGGACTGTGGT-3’
